# Risk of Major Adverse Cardiovascular Events and Venous Thromboembolism with JAK Inhibitors versus TNF Inhibitors in Rheumatoid Arthritis Patients: A Systematic Review and Meta-Analysis

**DOI:** 10.31138/mjr.171023.rof

**Published:** 2024-03-30

**Authors:** Styliani Partalidou, Dimitrios Patoulias, Kleopatra Deuteraiou, Paraskevi Avgerou, George Kitas, Maria Tzitiridou-Chatzopoulou, Theodoros Dimitroulas

**Affiliations:** 1 1^st^ Department of Internal Medicine, 424 Military Hospital of Thessaloniki, 56429, Thessaloniki, Greece; 2 Outpatient Department of Cardiometabolic Medicine, Second Department of Cardiology, Aristotle University of Thessaloniki, General Hospital “Hippokration”, 54642 Thessaloniki, Greece,; 3 4^th^ Department of Internal Medicine, Hippokration General Hospital, School of Medicine, Aristotle University of Thessaloniki, 54642, Thessaloniki, Greece,; 4 Research & Development, Dudley Group NHS Foundation Trust and University of Birmingham, United Kingdom

**Keywords:** JAK inhibitors, TNF inhibitors, rheumatoid arthritis, thromboembolic events, cardiovascular events

## Abstract

**Objective:**

The aim of this study was to compare the risk of major cardiovascular events (MACE) and venous thromboembolic events (VTE) between tumour necrosis factor (TNF) and Janus kinase (JAK) inhibitors in patients with rheumatoid arthritis (RA).

**Methods:**

We researched PubMed, Scopus, Cochrane Library, and clinicaltrials.gov until December of 2023 for randomised controlled trials (RCTs) and observational studies. The outcomes studied were MACE (stroke, heart attack, myocardial infarction, sudden cardiac death) and VTE (deep vein thrombosis, pulmonary embolism). We pooled data using random effects model. Risk for the reported outcomes was expressed as odds ratio (OR) with a 95% confidential interval (CI). We performed a subgroup analysis based on study design.

**Results:**

We identified 23 studies, 20 of which compared the odds for MACE and 14 the odds for VTE between JAK and TNF inhibitors in RA patients. Ten studies were RCTs and the rest were observational. Regarding MACE risk we pooled data from a total of 215,278 patients (52,243 were treated with JAK inhibitors, while the rest 163,035 were under TNF inhibitors). Compared with TNF inhibitors, the OR for JAK inhibitors in regards with MACE risk was 0.87 (0.64–1.17, p<0.01). Regarding VTE, a total of 176,951 patients were analysed (41,375 JAK inhibitors users and 135,576 TNF inhibitors users). The OR for VTE for JAK inhibitors compared with TNF inhibitors was 1.28 (0.89–1.84, p<0.01).

**Conclusion:**

According to our results, there is no statistically significant difference for MACE or VTE in RA patients who receive either JAK or TNF inhibitors.

## INTRODUCTION

Rheumatoid arthritis (RA) is an inflammatory disease affecting mainly small joints of hands, wrists and feet, usually in a symmetrical manner, but sometimes also affects larger joints and other systems.^1^ The main extra-articular manifestations include subcutaneous nodules, haematological abnormalities, such as anaemia or thrombocytosis, pulmonary involvement and secondary Sjögren’s syndrome.^2^ Moreover, RA patients have a greater risk for accelerated atherosclerosis and thromboembolic events compared to the general population due to a number of different factors, including the effects of systemic inflammation on vascular health and the higher prevalence of classical cardiovascular (CV) disease risk factors, such as hypertension, obesity, dyslipidaemia, and smoking, as well as the sedentary lifestyle.^3–5^ The increased CV risk results in significantly higher incidence of myocardial infarction, stroke or thromboembolic events in RA individuals compared to matched controls in population-based studies, while it accounts–amongst other co-morbidities - for the reduced life expectancy observed in this population.^6,7^

Efficient suppression of inflammation achieved with treat to target strategies and the administration of conventional and biologic disease modifying anti-rheumatic drugs (DMARDs) has significantly improved CV outcomes in RA patients.^8^ Over the last years, a new class of oral small molecules focused on Janus kinase (JAK) inhibition has been approved for the treatment of inflammatory arthropathies and for a variety of immune-mediated conditions, including inflammatory bowel disease (IBD), alopecia areata and atopic dermatitis. Despite the significant efficacy of JAK inhibitors in suppressing systemic inflammation several considerations have been raised about their use in RA, following the results of ORAL surveillance, a prospective head-to-head post-marketing phase IIIb–IV safety trial, which enrolled 4,362 patients with RA aged >50 years who had at least one cardiovascular risk factor. The results of the study showed an increased risk for CV and thromboembolic events in patients receiving tofacitinib – a non-selective JAK inhibitor – compared to those being treated with tumour necrosis factor (TNF)-alpha inhibitors adalimumab and etanercept.^9^ These findings have led regulatory agents, such as the United States Food and Drug Administration and the European Medicines Agency, as well as scientific associations, such as the European Alliance of Associations for Rheumatology (EULAR), to suggest limitations to the administration of JAK inhibitors for patients aged ≥65 years old, those with a history of smoking and those with risk factors for CV or thromboembolic disease.^10,11^ Given the efficacy of JAK inhibitors in the management of inflammatory conditions several observational studies have investigated the risk of CV and thromboembolic disease in RA patients receiving JAK inhibitors and other DMARDs in a real-world setting. Therefore, we performed a meta-analysis of both randomised controlled trials (RCTs) and observational studies to compare the odds for major CV and thromboembolic events in RA patients treated with JAK inhibitors and TNF-alpha inhibitors.

## MATERIALS & METHODS

The current study is a systematic review and meta-analysis that compares the odds for major cardiovascular events (MACE) and venous thromboembolic events (VTE) risk in adult patients with RA receiving either JAK inhibitors or TNF inhibitors. We planned to include both RCTs and observational studies. The JAK or TNF inhibitors could be any agent from the category that is approved for RA. More specifically, for JAK inhibitors we included baricitinib (BAR), upadacitinib (UPA), filgotinib, and tofacitinib (TOF). TNF inhibitors included infliximab, etanercept, adalimumab, certolizumab, and golimumab. We reported our results according to PRISMA guidelines.^12^

### Search strategy and inclusion criteria

We conducted our search in PubMed, Scopus, Cochrane Library, and Clinicaltrials.gov databases until 1^st^ December 2023. The research algorithm used was the following.

((JAK inhibitor*) OR (baricitinib) OR (tofacitinib) OR (filgotinib) OR (upadacitinib) OR (TNF alpha)) AND (rheumatoid arthritis) AND ((thrombos*) OR (thromboembolic) OR (cardiovascular event) OR (infarction) OR (stroke) OR (heart attack) OR (emboli*)). An English language filter was also applied.

In the meta-analysis we included patients with RA according to the 2010 ACR diagnostic criteria, aged at least 18 years old.^13^ Patients with IBD, psoriatic arthritis or any other disease that may use TNF or JAK inhibitors were not eligible.

### Data extraction

Data from the selected studies were exported from two independent investigators and included: author, publication year, utilised drug of interest, and dose-if reported (for JAK inhibitors and TNF inhibitors), total number of enrolled patients, number of patients with the outcome of interest, mean age of patients and major comorbidities, namely diabetes mellitus, cardiovascular disease, hypertension, history of stroke, and malignancy.

### Study outcomes

The composite outcomes studied were MACE, which included number of events of stroke, heart attack, myocardial infarction and sudden cardiac death, and VTE, which included deep vein thrombosis, and/or pulmonary embolism. No comparative efficacy endpoints were addressed in the present meta-analysis.

### Statistical analysis

The statistical analysis was conducted with Rstudio (version 2022.02.3). We conducted a meta-analysis comparing the events of MACE and VTE in JAK and TNF inhibitor groups. As they are binomial outcomes, we calculated odds ratios (ORs) along with 95% confidence intervals and applied an inverse-variance method for weighting studies. Next, we applied the DerSimonian-Laird estimator for calculating τ^2^ and we used a continuity correction of 0.5 in studies with zero cell frequencies. We extracted the OR both with random-effects and fixed effects model. However, we chose to present our results according to the first, due to various reasons. For example, random-effects model incorporates the heterogeneity, does not assume that all studies have identical intervention effects, and also, it awards more weight in smaller studies. For all the above, we assume that it is a more suitable approach for our study.^14^ Finally, we proceeded in subgroup analysis, based on the study design (RCT or observational study).

### Risk of bias assessment

The included studies were evaluated by two independent investigators regarding the risk of bias, using the Cochrane tools. We used ROB 2 and ROBINS-I tool for the randomised and non-randomised controlled trials respectively.^15,16^ Furthermore, we proceeded in assessment of the validity of outcomes, according to GRADE system.^17^

## RESULTS

### Study search results

**Figure 1.**
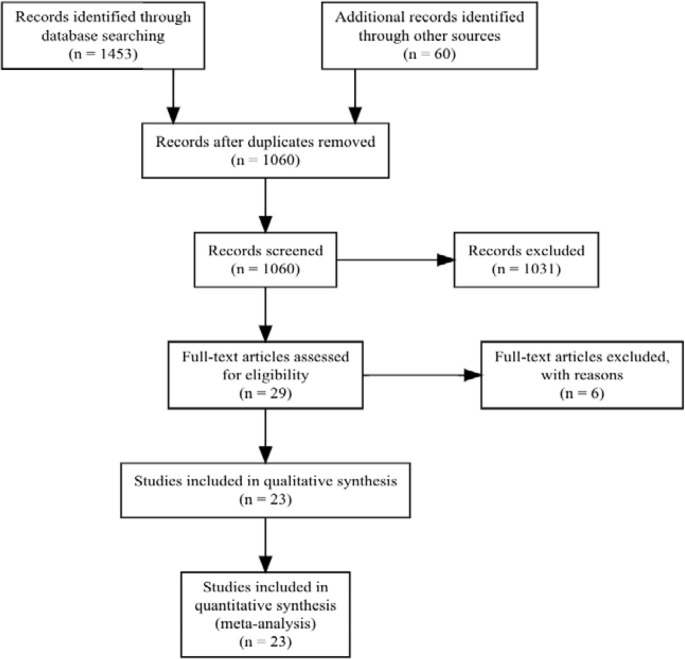
PRISMA flow diagram.

We conducted a thorough investigation of the literature and retrieved 1,453 articles according to our algorithm in PubMed (473), Scopus (745) and Cochrane Library (235). We, additionally, found 60 potentially eligible trials in ClinicalTrials.gov. After removal of the duplicates (459), 1,060 remained, which were assessed by two independent researchers at the level of title and abstract. Agreement was reached with a third investigator and 1,031 articles were excluded. Twenty-nine papers were then full-text assessed, and six of them were rejected, some because they did not report evidence for the studied adverse events (MACE, VTE), some trials were ongoing and no results have been posted yet, and finally some other studies compared JAK inhibitors with bDMARDs without discrimination for TNF inhibitors. PRISMA flow diagram depicting the study selection process is provided in **Figure 1**.^18^

We eventually included 23 studies in our meta-analysis, ten of which were RCTs, while the others were observational studies, mainly registries. Twenty studies, with 215,278 enrolled patients (JAK inhibitors=52,243, TNF inhibitors=163,035) compared odds for MACE between the two groups. Among these studies, nine of the eligible were RCTs. To compare the odds for VTE between JAK inhibitors and TNF inhibitors, we included 14 studies, six of which were RCTs. A total of 176,951 patients were analysed. Among them, 41,375 received a JAK inhibitor, while 135,576 were under TNF inhibitor treatment.^9,19–39^

### Study characteristics

Next, we present the main characteristics of the studies and patients in the studies included, along with the outcome of all the eligible studies (**Table 1**).

**Table 1.** Characteristics of included studies and patients.

**Study**	**Design**	**Agent**	**Comparator**	**Mean age**	**Comorbidities**	**Outcome (OR for MACE/VTE)**
**Bower, 2023**	RCT	All	all	59	Malignancy, DM, CKD, HF, VTE, CAD	0.95/−
**Burmester, 2023**	RCT	UPA	ADA	54	Stroke, HF, VTE, DM	1.33/1.33
**Cohen, 2020**	RCT, phase III	UPA	ADA	NR	NR	0.38/2.15
**Combe, 2020**	RCT, phase III	FIG	ADA	52.5	NR	0.62/2.63
**Deakin, 2023**	RCT	TOF	ADA	ADA: 56 TOF: 59	CKD	4.10/4.19
**Desai, 2019**	registry	TOF	All	1^st^ group 53, 2^nd^ group 71, 3^rd^ group 56	DM, AH, HF, AF, CAD, Stroke	−/0.95
**Fang, 2022**	registry	NR	NR	55.5	DM, AH, CKD, malignancy	0.96/0.78
**Fleischmann, 2019**	RCT, phase III	UPA	ADA	54	NR	0.14/0.50
**Frisell, 2022**	registry	BAR TOF	All	JAK: 60 TNF: 56.5	malignancy, infection, joint surgery, DM, ACS, stroke, COPD	0.57/−
**Hall, 2018**	RCT	TOF	ADA	56	NR	3.26/−
**Hong Ki Min, 2022**	observational	all	all	51.5	HT, DM CKD, HF	0.30/0.52
**Hoisnard, 2023**	registry	BAR TOF	ADA	59.3 55.3	NR	1.34/2.04
**Kohsrow-Khavar, 2021**	Registry	TOF	All	JAK: 60 TNF: 60	slightly higher CVD risk factors in TOF	1.05/−
**Mok, 2023**	Registry	All	All	53.8	JAK users had older age and more CVD risk factors	1.32/−
**Molander, 2023**	Registry	BAR TOF	All	JAK: 60 TNF: 57	Smoking, CKI, DM, COPD, Malignancy, stroke, surgery	−/1.49
**Song, 2023**	registry	all	all	NR	NR	0.86/0.95
**Salinas, 2023**	registry	BAR	All & Biosimilars	57.3	DM, AF, CAD, HF, AH, stroke	1.32/−
**Taylor, 2017**	RCT, phase III	BAR	ADA	53	NR	2.68/−
**Tong, 2023**	observational	BAR TOF UPA	all	52.7	AH, DM, CKD, malignancy, peripheral arterial disease, malignancy, COPD	0.86/−
**Uchida, 2023**	observational	BAR TOF	All	JAK: 67.5 TNF: 51	DM, COPD	4.40/−
**Wuslowska, 2022**	observational	BAR UPA TOF	All	TNF: 59.1 JAK: 61.7	NR	0.19/0
**Van Vollenhoven, 2012**	RCT	TOF	ADA	53	NR	0.33/−
**Ytterberg, 2022**	RCT	TOF	ADA etanercept	61.2	AH, DM, CVD	1.33/2.81

ADA: adalimumab; AF: atrial fibrillation; AH: arterial hypertension; BAR: baricitinib; CKD: chronic kidney disease; COPD: chronic obstructive lung disease; CAD: coronary artery disease; DM: diabetes mellitus; FIG: filgotinib; HT: hypertension; HF: heart failure; NR: not reported; RCT: randomized control trial; TNF: tumor necrosis factor; TOF: tofacitinib; UPA: upadacitinib.

### Risk of bias assessment

**Figure 2.**
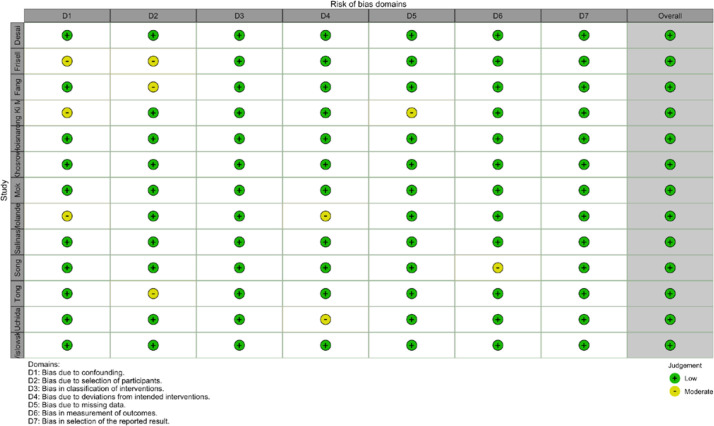
Bias assessment of non-randomised trials.

**Figure 3.**
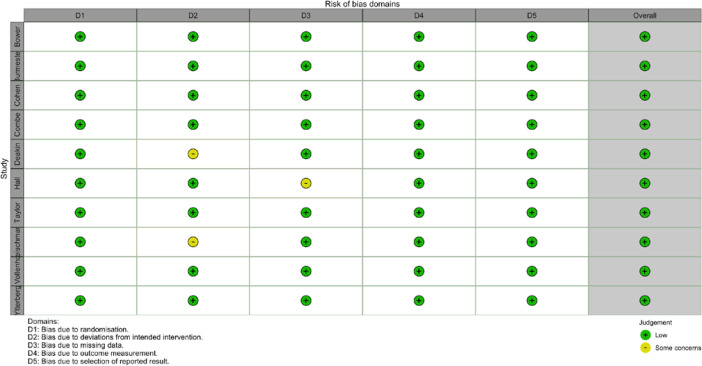
Bias assessment of randomised controlled trials.

Two independent investigators assessed the risk of bias for each eligible study. In case of disagreement, consensus was reached with a third investigator. As demonstrated by the diagrams below, no study was of low quality. RCTs were assessed via ROB-2 tool of Cochrane library, while observational studies were evaluated via ROBINS-I tool (**Figures 2 & 3**).^15,16^ Additionally, we present a summary of findings table according to GRADE recommendations (**Table 2**).^17^

**Table 2.** Summary of findings and GRADE assessment.

**Outcomes**	**Assumed risk TNFi use**	**Corresponding risk JAKi use**	**No of participants (studies)**	**Quality of evidence (GRADE)**	**Comments**
Major cardiovascular events (stroke, heart attack, myocardial infarction, sudden cardiac death)	5.332 events in a total of 162.462 patients, from 20 studies, nine of which are RCTs (high quality)	844 events in a total of 52.243 patients, from 20 studies (nine RCTs)	214.705 (20)	high	Despite the low quality that observational studies have by definition, the OR is similar in the subgroup analysis, indicating high quality in other fields
Venous thromboembolism events (deep venous thrombosis, pulmonary embolism)	971 events in a total of 135.576, from 14 studies, six of which are RCTs (high quality)	406 events in a total of 29.974, from 14 studies (six RCTs)	165.550 (14)	moderate	Although the result is non-significant both in the subgroup analysis and in total, the OR in the RCT group is slightly higher

Patient or population: patients with rheumatoid arthritis

Settings

Intervention: use of JAK inhibitors (any agent)

Comparison: use of TNF inhibitors (any agent)

OR: odds ratio; RCT: randomised controlled trial

### Comparison of MACE risk between JAK and TNF groups

**Figure 4.**
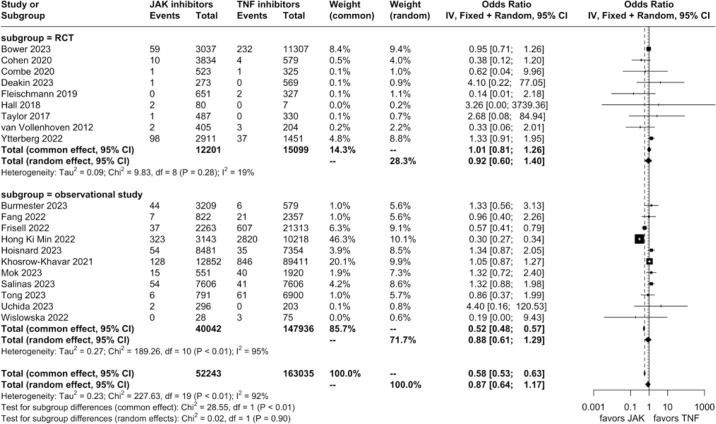
Comparison of major cardiovascular events risk between JAK and TNF inhibitors users among rheumatoid arthritis patients.

We proceeded in a meta-analysis comparing odds for MACE between the two groups. The OR for JAK inhibitors compared to TNF inhibitors was 0.87 (CI: 0.64–1.17, p<0.01). Also, the I^2^ index was 92%, indicating high heterogeneity.^14^ Taking a closer look in the subgroup analysis, though, the heterogeneity index is much less in the RCT subgroup, despite that the OR is similar to the total result. Fleischmann et al. and Wislowska et al. studies seem graphically to present the most favourable outcome for JAK inhibitors agents. Specifically, Fleischmann et al. compared adalimumab and UPA, while patients were also under standard dose of MTX and on demand use of non-steroid anti-inflammatory drugs or low dose glucocorticoids. On the other hand, Wilsowska et al. enrolled patients receiving any TNF inhibitor compared with BAR, UPA or TOF from the JAK inhibitor arm. They were, also, under MTX at the same time.^21,27^ With the subgroup analysis, based on the study design, the OR for JAK in the RCTs is quite similar compared to the observational studies (**Figure 4**).

### Comparison of VTE risk between JAK and TNF group

**Figure 5.**
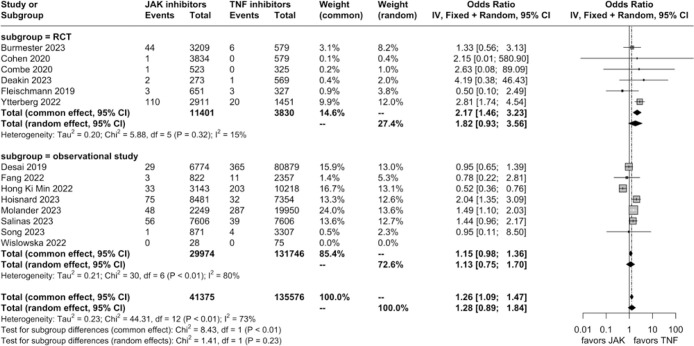
Comparison of venous thromboembolism between JAK and TNF inhibitors users among rheumatoid arthritis patients.

We also conducted a meta-analysis about odds for VTE among RA patients under either JAK or TNF inhibitors. The OR of JAK inhibitors in comparison with TNF inhibitors was 1.28 (CI: 0.89–1.84, p<0.01). The heterogeneity in this analysis was considered as high, reaching up to 73%. Again, as in MACE meta-analysis, the heterogeneity in the RCT group is much lesser, up to 15%. In this meta-analysis, the OR in the RCT subgroup is slightly higher, yet non-significant (**Figure 5**).

## DISCUSSION

In this meta-analysis we compared MACE and VTE odds among patients with RA, taking either TNF inhibitors or JAK inhibitors. According to our results, OR for MACE with JAK inhibitors versus TNF inhibitors was 0.87 (CI: 0.64–1.17, p<0.01), while OR for VTE risk was 1.28 (CI: 0.89–1.84, p<0.01). Our findings suggest that neither MACE nor VTE have statistically significant difference between the two groups. Furthermore, our subgroup analysis indicated that in RCTs the OR of JAK inhibitors compared with TNF for the outcomes studied is similar compared to the observational studies, despite the lower heterogeneity RCT subgroups displayed. Given that RCTs are of utmost credibility, we could assume that these results may reflect the similar risk that JAK inhibitors oppose for MACE and VTE in real life clinical setting. The findings of our analysis concur with some, but not all, published data suggesting that the risk of MACE and thromboembolic events in patients with immune mediated diseases under JAK inhibitors is not increased compared to those receiving TNF inhibitors.^23,40,41^ It appears that the major concern regarding the use of JAK inhibitors is the risk of venous thromboembolism, as indicated by a comparative cohort study between RA individuals initiating treatment with JAK inhibitors or other biologic DMARD.^30,42^ On the other hand, a couple of recently published post hoc analyses of the ORAL surveillance study have identified specific TOF treated subpopulations at higher risk for MACE and thromboembolic events compared to TNF inhibitors. More specifically, age ≥65 years and smoking were demonstrated to be independent risk factors, as over 80% of MACE or thromboembolic events in TOF-treated patients were attributable to a combination of these two factors. In addition, increased risk for MACE and VTE was observed only in patients with a history of atherosclerotic CV or thromboembolic disease, respectively. These findings highlight the complexity of CV risk in RA and the importance of assessing overall CV and other certain risk factors, when considering JAK inhibitors as treatment option in RA patients. However, it should be noted that this therapeutic dilemma on whether to initiate JAK inhibitors or TNF inhibitors is placed at the second step in the EULAR’s recommendations for the management of RA, as MTX remains the mainstay of initial management.^11^

Our findings are also in line with another large recent registry, which assessed the safety of UPA in patients with RA and other immune diseases. Burmester et al. found neither increased MACE nor VTE with the use of UPA, compared to ADA. The patients that presented some of these adverse events, had already increased cardiovascular and/or thromboembolic risk. The most common event was pulmonary embolism, which is, however, increased in patients with RA, independently of JAK inhibitor use.^5,43,44^ Furthermore, one recent meta-analysis of RCTs examining the risk of VTE with JAK inhibitors in various immune-mediated diseases compared to placebo, did not find a significant difference, as well.^45^ In agreement with the above statement is also the real-world data cohort in Khosrow-Khavar et al. study, which is included in our meta-analysis. They found that MACE risk in patients under JAK inhibitors was increased only in those who had already advanced cardiovascular risk.^24^ Another large study with RA patients treated with TOF, also, states that classic risk factors are associated with increased risk for MACE in this population.^46^

However, the findings of several studies are conflicting. For instance, a case-series study, also, demonstrated higher risk for MACE and VTE in patients treated with BAR or TOF, which was sustained for about a month after drug discontinuation.^47^ Additionally, a ten-year pharmacovigilance study, based on FDA reporting system for adverse events, highlighted the high risk for thromboembolic events with JAK inhibitors.^48^

It is obvious that despite the wide use of these agents, and accumulating data, this topic remains debating. Until it is clear, it is preferable to consider the patients’ CV risk status before initiating a JAK inhibitor. In this regard, a scientific group from France recently released some recommendations to assist the decision on initiating JAK inhibitors in patients with high CV or thromboembolic risk.^49^ EULAR, also recommends avoiding, if possible, and preferring other agents in high CV risk patients for the management of RA.^11^

One of our analysis’ strengths is that it includes a large number of trials and patients. What is important is that it showed similar risk for experiencing MACE or VTE in RA patients treated either with JAK inhibitors or TNF inhibitors. Finally, it underlines that the results between large-scaled RCTs and smaller observational studies appear to be comparable.

One of the limitations of the current study is the absence of a sensitivity analysis regarding the dose of each agent used, as the outcomes of interest may be dose-dependent. Second, the absence of linear regression and stratification of the enrolled patients according to various classic CV risks appears to be additional limitation.

## CONCLUSION

According to our meta-analysis, there is no statistically significant difference in the odds for MACE and VTE among patients with RA treated with either TNF inhibitors or JAK inhibitors. Due to conflicting data regarding this topic in the literature, it is better to assess the CV risk of each patient prior to treatment initiation and avoid the use of JAK inhibitors in patients featuring high CV risk. Larger head-to-head clinical trials are needed to shed additional light on this controversial topic, especially in high-CV risk population.

## Data Availability

The data supporting the conclusions of this article are in this article.

## ABBREVIATIONS

BAR: baricitinib
CV: cardiovascular
DMARD: disease modifying anti-rheumatic drugs
EULAR: European Alliance of Associations for Rheumatology
GCs: corticosteroids
IBD: inflammatory bowel disease
JAK: Janus kinase
MACE: major adverse cardiovascular events
MTX: methotrexate
RA: rheumatoid arthritis
RCT: randomised controlled trial
TNF: tumour necrosis factor
TOF: tofacitinib
UPA: upadacitinib
VTE: venous thromboembolism
